# Bringing collections out of the dark

**DOI:** 10.3897/zookeys.209.3699

**Published:** 2012-07-20

**Authors:** Vincent S. Smith, Vladimir Blagoderov

**Affiliations:** 1 Natural History Museum, Cromwell Road, London, SW7 5BD, U.K.

Natural history collections are an incomparable treasure and source of knowledge. Collected over centuries of field exploration, these repositories contain a sample of the world’s biodiversity, and represent a monumental societal investment in research and applied environmental science (Network Integrated Biocollections Alliance 2010). Knowledge derived from the 1.5–3 billion specimens (Ariño 2010, Duckworth et al. 1993) within these collections has made vital contributions to the study of taxonomy, systematics, invasive species, biological conservation, land management, pollination and biotic responses to climate change (Chapman 2005). Despite these activities, natural history collections are significantly underutilised due to the difficulty of obtaining and analysing data within and across collections. Digitisation and mobilisation of specimen and associated data removes this impediment, but presents major technical and organisational challenges. The largest of these is how to capture specimen data fast enough to achieve digitisation of entire collections while maintaining sufficient data quality.

Until recently, episodic and incremental funding has had limited success with natural history digitisation, largely addressing local projects within single institutions or across niche research communities. New funding, coupled with more collaborative approaches to digitisation, and technical advances with scanning and imaging systems have begun to change this. The collection of eighteen articles published here examines some of these developments, providing a snapshot of current digitisation efforts and progress across these themes.

The first of these papers by Reed Beaman and Nico Cellinese (2012) looks at the transformative potential of natural history specimen digitisation, both in terms of driving new developments in technical infrastructure, as well as in new applications for the digitised products of this work. Fundamental to the increase in efficiency of these programmes is the modularisation of the digitisation process. Collections digitisation is broadly defined to include transcription into electronic format of various types of data associated with specimens, the capture of digital images of specimens, and the georeferencing of specimen collecting localities. These steps are examined by Gill Nelson and colleagues (2012), who are quite literally based at the ‘hub’ of National Science Foundation efforts to advance the digitisation of North American biological collections in the United States. Based on studies of major digitisation efforts across the U.S., Nelson et al. break down the clusters of digitisation activities into workflows that can be adopted by other digitisation efforts.

A fundamental step in any digitisation programme is the aggregation or federation of digital output so it can be collectively searched and discovered. The European Union funded *Open-UP* project is one such effort within Europe, and is described by Anton Güntsch and Walter Berendsohn (2012) in their paper on the mobilisation of natural history multimedia resources through the *EUROPEANA* data portal. The challenges surrounding the coordination of digitisation efforts are also looked at through a series of projects trying to address these problems, nationally or via thematic networks. In some cases these are best practice networks such as the *U.S. Virtual Herbarium* described by Mary Barkworth and Zack Murrell (2012). In other cases these projects provide a service infrastructure such as the Finnish *Digitarium* (Tegelberg et al. 2012). Even operating within the confines of a single large institution can be a challenge: different stakeholders have different priorities that can be difficult to accommodate within the budgets of single institutions. Marc Gofferjé and Jon Peter van den Oever (2012) describe a range of solutions to address these issues at NCB Naturalis. Part of the solution lies in improving the efficiency of an institutions digitisation process, as illustrated at the New York Botanic Gardens (Tulig et al. 2012) and the Royal Botanic Gardens Edinburgh (Haston et al. 2012).

Attempts to automate digitisation are confounded by the fact that different types of organisms require very different types of preservation. Plants and fungi are typically prepared as dried, flattened specimens attached to archival quality paper, with printed label data mounted on the sheet. This pre-adapts herbaria to rapid digitisation. In contrast insects, which are the most numerous organisms in collections, are typically mounted by pinning individuals on entomological pins, which are accompanied by tiny (often folded) labels beneath each specimen. The particular demands of mass digitising entomological specimens are the subject of five papers, which have methodologically converged on the scanning whole collection drawers. *GigaPan*, described by Matthew Bertone and colleagues (2012) was arguably the first of these approaches, enabling the low cost capture of gigapixel panoramas of insect museum drawers containing many hundreds of specimens. More recently *SatScan*, developed in association with the Natural History Museum London (Blagoderov et al. 2012), and in use at the Australian National Insect Collection (Mantle et al. 2012) has enabled these panoramas to be obtained with minimal distortion. *SatScan* is accompanied by software used to select and annotate images of individual specimens. The drawer scanning approach has been incorporated as part of the U.S. *InvertNet* digitisation programme (Dietrich et al. 2012), and has resulted in a new, low cost instrument called *DScan* (Schmidt et al. 2012). A contrasting approach to accessing digital images is described by Quentin Wheeler and colleagues (2012), who are exploring the use of telemicroscopy to enable remote researchers to access and manipulate specimens beyond their physical reach. Although not strictly mass digitisation, the potential effect of this network of remote access microscopes is similar, enabling researchers to examine insect material located at major institutions over a network connection.

Even with this automation, a significant labour force is still critical for many digitisation projects. Paul Flemons and Penny Berents (2012) explore the use of volunteers to increase the rate of digitising insect collections. This has enabled the Australian Museum to capture label data and images for 16,000 specimens in just 5 months. Label data transcription is a major problem in many digitisation projects. Andrew Hill and colleagues (2012) describe their software to crowdsource label transcription through a workforce of citizen scientists. Embedding quality control techniques and design elements to keep contributors motivated, *Notes On Nature* provides a toolkit for transcription of ledgers and labels of natural history specimens. Andrea Thomer and colleagues (2012), extend this transcription work into new territory using Wiki-style templates to crowdsource data extraction from century-old field notebooks. This enables interoperability of the underlying data without losing the narrative context from which these observations are drawn. The series closes with a paper by Randall Schuh (2012), who looks at methods to integrate specimen databases into the practice of revisionary systematics, closing the loop between digitising, extracting and reusing data in taxonomic research.

In bringing together this special issue on digitisation we have sought to represent a wide selection of projects and techniques. These papers provide a snapshot of activity in what is a fast moving field that is seeing ever-increasing degrees of collaboration across disciplines and between collection-based institutions. Many of these projects deal with the unique challenges associated with major collections that have built up over several centuries, with different communities of practice and different user groups. Despite these differences, the standards for collection acquisition, preservation and documentation are broadly consistent, meaning that there is sufficient common ground to bring together the enormous amounts of data that are being exposed through these activities. We expect that in the next decade these data will become the new frontier for natural history collection management and research.
